# Direct Preparation of Alginate Oligosaccharides from Brown Algae by an Algae-Decomposing Alginate Lyase AlyP18 from the Marine Bacterium *Pseudoalteromonas agarivorans* A3

**DOI:** 10.3390/md22110483

**Published:** 2024-10-26

**Authors:** Xiao-Hui Sun, Xiao-Dong Zhang, Xin-Ru Zhang, Xiao-Fei Wang, Xi-Ying Zhang, Yu-Zhong Zhang, Yu-Qiang Zhang, Fei Xu

**Affiliations:** 1 State Key Laboratory of Microbial Technology, Marine Biotechnology Research Center, Shandong University, Qingdao 266237, China; sxh19961219@163.com (X.-H.S.); 202312555@mail.sdu.edu.cn (X.-D.Z.); 202232620@mail.sdu.edu.cn (X.-R.Z.); 202212539@mail.sdu.edu.cn (X.-F.W.); zhangxiying@sdu.edu.cn (X.-Y.Z.); zhangyz@sdu.edu.cn (Y.-Z.Z.); 2 Joint Research Center for Marine Microbial Science and Technology, Shandong University and Ocean University of China, Qingdao 266237, China; 3 Frontiers Science Center for Deep Ocean Multispheres and Earth System & College of Marine Life Sciences, Ocean University of China, Qingdao 266237, China; 4 Shandong Academy of Grape, Shandong Academy of Agricultural Sciences, Jinan 250199, China

**Keywords:** alginate lyase, alginate oligosaccharides, brown algae, *Laminaria japonica*, *Macrocystis pyrifera*

## Abstract

Alginate oligosaccharides (AOs), derived from alginate degradation, exhibit diverse biological activities and hold significant promise in various fields. The enzymatic preparation of AOs relies on alginate lyases, which offers distinct advantages. In contrast to the conventional use of sodium alginate derived from brown algae as the substrate for the enzymatic preparation of AOs, AO preparation directly from brown algae is more appealing due to its time and energy efficiency. Thus, the identification of potent alginate lyases and cost-effective brown algae substrates is crucial for optimizing AO production. Herein, we identified and characterized an alginate lyase, AlyP18, capable of efficiently decomposing algae, from a marine bacterium *Pseudoalteromonas agarivorans* A3 based on secretome analysis. AlyP18 is a mesothermal, endo-type and bifunctional alginate lyase with high enzymatic activity. Two brown algae substrates, *Laminaria japonica* roots and *Macrocystis pyrifera*, were used for the AO preparation by AlyP18. Upon optimization of AlyP18 hydrolysis parameters, the substrate degradation efficiency and AO production reached 53% and ~32% for *L. japonica* roots, respectively, and 77% and ~46.5% for *M. pyrifera*. The generated AOs primarily consisted of dimers to pentamers, with trimers and tetramers being dominant. This study provides an efficient alginate lyase and alternative brown algal feedstock for the bioconversion of high-value AOs from brown algae.

## 1. Background

Brown algae are highly productive algae in the ocean and have attracted increasing attention due to their high contents of polysaccharides, rapid growth rate and the absence of recalcitrant lignin [1,2,3]. Several species of brown algae, including *Laminaria*, *Macrocystis* and *Sargassum* sp., are well-known for their large biomass production [4,5,6].

Alginate is the most abundant polysaccharide in the cell walls of brown algae, accounting for ~40% of cell dry weight [7]. Alginate consists of β-D-mannuronate (M) and its C-5 epimer α-L-guluronate (G), which exist as blocks such as polyM (PM), polyG (PG) and polyMG/GM (PMG) [8]. The monomeric ratios (M/G) of alginate vary among species of brown algae [7]. Alginate is known to be widely used as a hydrocolloid in various applications, such as biomedicine and pharmacy [9], and its degradation products, alginate oligosaccharides (AOs), are gaining increasing attention for their beneficial roles in pharmaceuticals, food and agricultural industries [10]. The present ways to produce AOs include physical, chemical and enzymatic methods [11]. Among these, enzymatic methods, mainly through alginate lyases, have the advantages of high efficiency, mild degrading conditions and environmental friendliness [12,13]. Therefore, alginate lyases that efficiently generate AOs have great application prospects and have sparked renewed interest.

Alginate lyases catalyze the degradation of alginate via the β-elimination mechanism, cleaving 1,4 glycosidic bonds and generating unsaturated double bonds at the non-reducing end [14]. According to the action mode, alginate lyases can be grouped into endo-type and exo-type enzymes. Endo-type alginate lyases depolymerize alginate into AOs, while exo-type ones remove monomers or dimers one by one from the ends of alginate [15]. Based on the substrate specificities, alginate lyases can be categorized into PM-specific lyases (EC 4.2.2.3), PG-specific lyases (EC 4.2.2.11) and bifunctional lyases that can catalyze both PM and PG degradation (EC 4.2.2.-) [16]. In the Carbohydrate-Active enZYmes (CAZy) database, alginate lyases are classified into 16 polysaccharide lyase (PL) families (PL5, 6, 7, 8, 14, 15, 17, 18, 31, 32, 34, 36, 38, 39, 41 and 44) [17]. Many recent discoveries of alginate lyases have been on new PL7 enzymes derived from *Flavobacterium* spp. and PL5 enzymes from *Sphingomonas* spp. [16].

Most studies on AO preparation have focused on the screening and characterization of alginate lyases for the efficient degradation of sodium alginate [12]. However, the extraction of sodium alginate from brown algae requires multiple steps [7]. Compared with sodium alginate, using brown algae as raw material to produce AOs is more eco-friendly, convenient and cost-effective. However, till now, only two alginate lyases, AlgL7 and VfAly7, have been reported to produce AOs directly from brown algae [18,19]. Moreover, only a limited number of brown algae substrates, such as *Laminaria japonica* and *Undaria pinnatifida*, have been used for AO production by alginate lyases [18,19]. Therefore, the identification of more alginate lyases capable of efficiently producing AOs directly from brown algae and the exploration of viable brown algae feedstock is of great significance, which avoids the complicated process of extracting alginate from brown algae, saving time and reducing contamination.

*Pseudoalteromonas agarivorans* A3, screened from a *L. japonica* sample by our lab, has been demonstrated as a *L. japonica*-decomposing strain by secreting alginate lyases [20]. Here, we identified the dominant alginate lyase AlyP18 secreted by strain A3, which may play an essential role in the decomposition of *L. japonica*. The *alyP18* gene was overexpressed in *Escherichia coli,* and the recombinant protein AlyP18 was purified and characterized. Moreover, we evaluated the potential of AlyP18 in preparing AOs directly from brown algae, including *Macrocystis pyrifera* and the roots of *L. japonica*. The results indicate that AlyP18 is a promising alginate lyase for efficient AO production directly from brown algae, which may have potential applications in industry.

## 2. Results and Discussion

### 2.1. Identification of the Most Abundant Alginate Lyase, AlyP18, Secreted by Strain A3

*P. agarivorans* A3, isolated from a fresh *L. japonica* sample was previously screened by our lab for its remarkable ability to decompose *L. japonica* by secreting alginate lyases [20]. To identify the most efficient alginate lyase(s) produced by strain A3 for *L. japonica* decomposition, the secretome of strain A3 cultivated under the optimum conditions for alginate lyase production was analyzed [20]. Consequently, three putative alginate lyases (AlyP18, AlyP6 and AlyP7) belonging to three different PL families were detected (Table 1). Among them, the abundance of the PL18 AlyP18 (95.09%) outdistanced that of the other two lyases, suggesting that AlyP18 may play a significant role in the degradation of *L. japonica* by strain A3.

### 2.2. Sequence Analysis of the Alginate Lyase AlyP18

**Figure 1:**
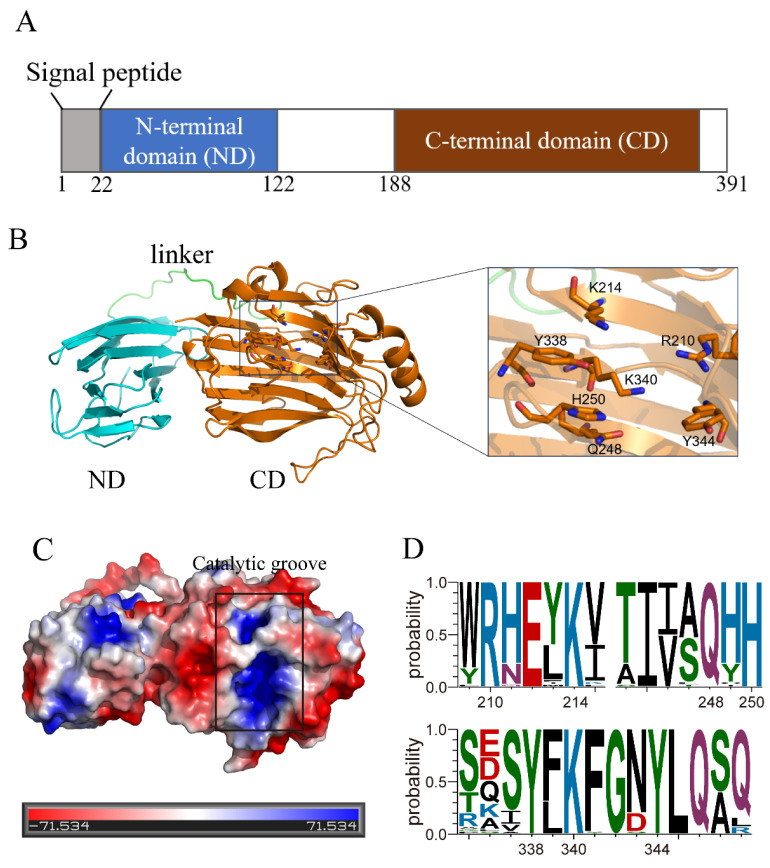
Sequence and structure analyses of the alginate lyase AlyP18. (**A**) Schematic domain diagram of AlyP18 annotated by the NCBI’s Conserved Domain Database. (**B**) The overall structure of AlyP18 predicted by Alphafold2. The residues are presented as sticks. (**C**) Surface electrostatic potential of AlyP18. The predicted catalytic groove is boxed. The figure was prepared using PyMOL. (**D**) Profile of conserved residues that participated in catalysis and substrate recognition of PL18 alginate lyases. Residues are numbered in accordance with AlyP18. WebLogo (http://weblogo.threeplusone.com, accessed on 25 September 2024) was used to create the sequence logos.

The AlyP18-encoding gene *alyP18* is 1176 bp in length. AlyP18 consists of 391 amino acid residues, including a predicted signal peptide of 22 residues (Met1–Ala22) (Figure 1A). According to the Conserved Domain analysis, AlyP18, akin to other PL18 enzymes, is comprised of two domains, an N-terminal domain (ND) and a C-terminal domain (CD), connected by a linker (Figure 1A) [21,22]. The ND of the PL18 alginate lyase Aly-SJ02 has been identified as an intramolecular molecular chaperone, ensuring the proper folding of the CD. The CD of Aly-SJ02 functions as the catalytic domain responsible for alginate degradation [21]. The CD and ND in AlyP18 may play similar roles to those observed in Aly-SJ02. Sequence analysis revealed that AlyP18 shared an identical sequence with the endo-type alginate lyase AlyA from *P. atlantica* AR06 [23]. However, only the CD of AlyA (residues 148−391 or 162−391 of AlyA) obtained from the culture supernatant of strain AR06 has been studied [23].

Though the CD structure of the PL18 alginate lyase has been revealed [21], the structure of the full-length protein remains to be explained. The predicted AlyP18 structure revealed that the ND (residues 23–146) featured a β-sandwich fold with eleven antiparallel β-strands arranged into two distinct sheets, while the CD (residues 166–391) adopted a β-jelly roll structure primarily composed of two antiparallel β-sheets (Figure 1B). ND and CD are linked by a 19-residue extended coil (residues 147–165), thus ensuring the positively charged catalytic groove of CD is fully exposed (Figure 1B,C). The catalytic groove is open at both ends (Figure 1C), implying an endolytic action mode. The conserved residues, including Tyr344, Arg210, Lys214, Gln248, His250, Tyr338 and Lys340, were observed in the catalytic groove of AlyP18 (Figure 1B,D), which may be responsible for catalysis or substrate recognition, similar to their roles in other PL18 alginate lyases [21].

### 2.3. Biochemical Characterization of the Alginate Lyase AlyP18

**Figure 2:**
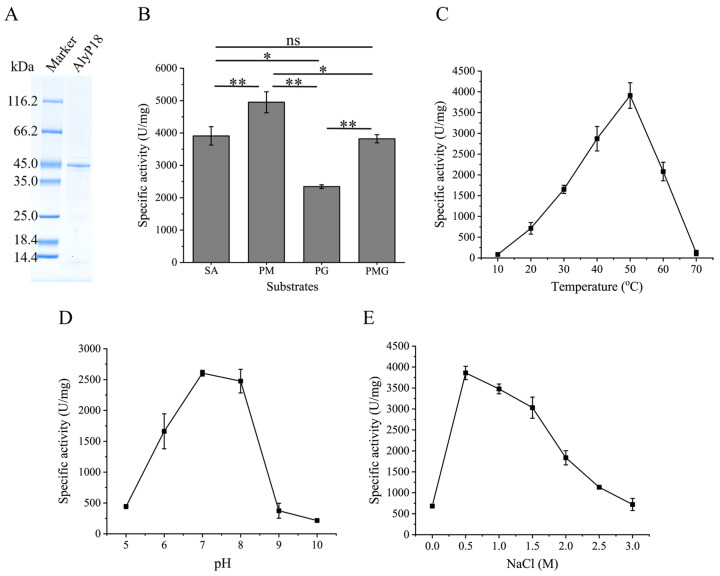
Effects of reaction conditions on AlyP18 activity. (**A**) SDS-PAGE analysis of the purified AlyP18. (**B**) Substrate specificity of AlyP18. The reaction mixture containing 2 mg/mL substrates, 50 mM Tris-HCl (pH 7.0), 0.5 M NaCl and 7.5 nM AlyP18 was conducted at 50 °C for 15 min. SA, sodium alginate. *, **, ns: significant at *p* < 0.05, 0.01, or not significant, respectively. (**C**) Effect of temperature on AlyP18 activity. Experiments were performed in 50 mM Tris-HCl buffer (pH 7.0) containing 0.5 M NaCl at temperatures ranging from 10 °C to 70 °C for 15 min. (**D**) Effect of pH on AlyP18 activity. Experiments were conducted in Britton–Robinson buffer with a pH range of 5.0 to 10.0 and 0.5 M NaCl at 50 °C for 15 min. (**E**) Effect of NaCl concentration on AlyP18 activity. Experiments were performed in 50 mM Tris-HCl buffer (pH 7.0) and NaCl at concentrations from 0 to 3.0 M at 50 °C for 15 min. Sodium alginate was used as the substrate in assays (**C**–**E**). The data presented in (**B**–**D**) are from triplicate experiments (mean ± standard deviation [SD]).

The gene *alyP18* without the predicted signal peptide sequence was intracellularly overexpressed in *E. coli* (DE3). The resulting recombinant alginate lyase AlyP18, comprising both the ND and CD, was successfully purified. It exhibited an apparent molecular mass of approximately 44 kDa based on SDS-PAGE analysis (Figure 2A), which was in accordance with the theoretical value of 42.43 kDa. Although several PL18 alginate lyases have been documented, their characterization mainly focused on their CDs rather than the full-length proteins. Specifically, only the CDs of the alginate lyase from *Alteromonas* sp. 272 and AlyA were isolated through protein purification from their respective fermentation cultures [24,25]. Additionally, the CDs of the PL18 alginate lyases AlyPEEC and Aly-SJ02 were heterologously expressed [21,22]. Differently, we expressed AlyP18, which comprises both the ND and CD in *E. coli*, which will contribute to the comprehensive understanding of the PL18 alginate lyases.

AlyP18 displayed lytic activity towards various alginate substrates, including PM, PG, PMG and sodium alginate (Figure 2B), demonstrating that it is a bifunctional alginate lyase, consistent with the other characterized PL18 alginate lyases [21,23,24]. AlyP18 exhibited the maximum activity at 50 °C, indicating that it is a mesothermal alginate lyase (Figure 2C). Due to their marine origin, most reported alginate lyases are cold-adapted enzymes, with enzyme activity reaching a maximum at temperatures below 35 °C and a sharp drop above 40 °C [25,26,27]. Many efforts have been made to improve their reaction temperature. This is because a high reaction temperature (above 45 °C) can promote substrate transformation by decreasing the viscosity of alginate and can prevent microorganism contamination when processing crude substrate, such as kelp powder [2]. Therefore, AlyP18 with a high catalytic temperature is highly valuable in industry. Additionally, AlyP18 showed optimal activity at pH 7.0 and retained 95% activity at pH 8.0 (Figure 2D). NaCl at 0.5 M increased AlyP18 activity by 5.6-fold compared to NaCl at 0 M. Even in the presence of 3 M NaCl, AlyP18 exhibited higher activity than at 0 M NaCl, suggesting that the activity of AlyP18 was activated by NaCl and that AlyP18 exhibited salt tolerance (Figure 2E). Most marine-derived alginate lyases show an optimal pH of 7.0–8.0 and are activated by NaCl. For example, the PL7 alginate lyase AlyC3 showed an optimal pH and NaCl concentration at 8.0 and 0.5 M, respectively [28]. Another PL7 alginate lyase, AlgNJ-04, exhibited the optimal pH and NaCl concentration at 7.0 and 0.5 M [29]. In addition, compared to most characterized alginate lyases, which have a specific activity below 2000 U/mg, such as the PL7 alginate lyase Alyw201 and the PL17 alginate lyase Alg17B [30,31], AlyP18 is a highly active enzyme with an activity of 3911.05 U/mg (determined by the ultraviolet absorption method) towards sodium alginate under the optimal reaction conditions. Meanwhile, the enzyme activity of AlyP18 was determined by the dinitrosalicylic acid (DNS) method with 59.86 ± 4.72 U/mg. Furthermore, the *K*_m_ value of AlyP18 toward sodium alginate was determined to be 0.46 ± 0.04 mg, lower than that of most reported alginate lyases [15], indicating that AlyP18 has a high affinity for sodium alginate.

**Figure 3:**
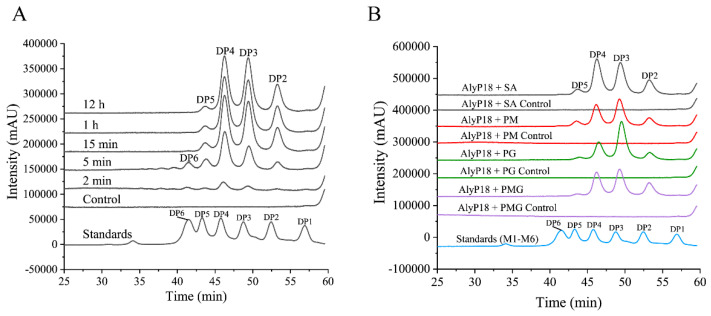
Analysis of the alginate degradation products by AlyP18. (**A**) Time−course analysis of the degradation of sodium alginate by AlyP18. AlyP18 was incubated with 2 mg/mL sodium alginate under the optimum reaction conditions for 2 min, 5 min, 15 min, 1 h or 12 h, and the resulting products were analyzed by HPLC. The control was performed with the pre-heated inactivated lyase. (**B**) HPLC analysis of the final degradation products of AlyP18 toward different substrates. AlyP18 was incubated with 2 mg/mL substrates under the optimum reaction conditions for 12 h. The standards used were mannuronate oligomers ranging from DP1 to DP6. DP, degree of polymerization. SA, sodium alginate. The figures are representatives of three experimental repeats.

To investigate the degradation pattern of AlyP18, AlyP18 was reacted with sodium alginate at 50 °C for 2 min, 5 min, 15 min, 1 h and 12 h, respectively. The degradation products of AlyP18 at different times were AOs with degrees of polymerization (DP) ≥ 2, demonstrating that AlyP18 is an endo-type alginate lyase (Figure 3A), which is consistent with the structural analysis (Figure 1C). With different types of alginate (sodium alginate, PM, PG and PMG) as the substrates, AlyP18 consistently produced disaccharides, trisaccharides, tetrasaccharides and pentasaccharides as the final degradation products, with trimers and tetramers being predominant (Figure 3B). These results suggested that AlyP18 had great potential in preparing AOs with DP2–DP5.

### 2.4. Optimization of the Hydrolysis Parameters of AlyP18 on L. japonica Roots and M. pyrifera

Considering the potential significance of AlyP18 in *L. japonica* decomposition by strain A3 and its excellent enzymatic characteristics, we speculated that AlyP18 had a promising prospect in directly preparing AOs from brown algae. *L. japonica*, a vital primary producer in coastal oceans, is extensively cultivated as an edible brown alga [32]. In food processing, its meristem and stipe, colloquially referred to as the “roots”, are often discarded, resulting in resource wastage. *M. pyrifera*, the largest and most widely distributed kelp species, is commonly harvested for extracting phycocolloids, such as alginate, owing to its high content (~70%) of polysaccharides [5,33,34]. Hence, we used *L. japonica* root powder and *M. pyrifera* powder as raw materials and optimized four parameters for the hydrolysis of AlyP18 on them, including the enzyme–substrate ratio (E/S ratio), hydrolysis time, hydrolysis temperature and hydrolysis pH. Due to the diminished enzyme activity of AlyP18 at pHs below 7.0 and above 8.0 (Figure 2D), we exclusively analyzed hydrolysis on the two substrates at pH 7.0 and pH 8.0.

**Figure 4:**
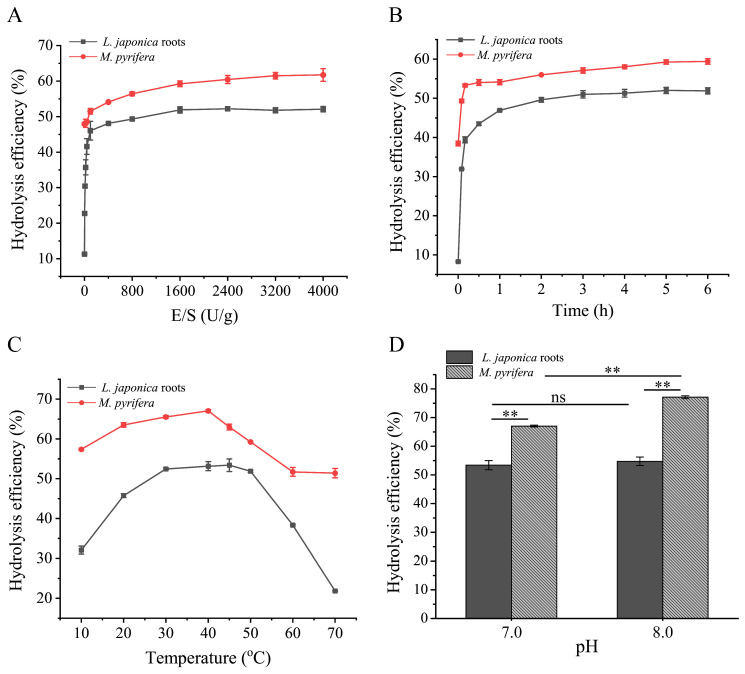
Hydrolysis of *L. japonica* roots and *M. pyrifera* by the alginate lyase AlyP18. (**A**) Effect of E/S ratio on the hydrolysis efficiency. Hydrolysis of AlyP18 on *L. japonica* roots and *M. pyrifera* was conducted at 50 °C and pH 7.0 with different E/S ratios. (**B**) Effect of hydrolysis time on the hydrolysis efficiency. AlyP18 was incubated with *L. japonica* roots and *M. pyrifera* at 50 °C and pH 7.0 for different times with an E/S ratio of 1600 U/g for *L. japonica* roots and 3200 U/g for *M. pyrifera*. (**C**) Effect of hydrolysis temperature on the hydrolysis efficiency. AlyP18 was incubated with *L. japonica* roots and *M. pyrifera* at various temperatures ranging from 10 °C to 70 °C at pH 7.0. (**D**) Effect of hydrolysis pH on the hydrolysis efficiency. AlyP18 was incubated with *L. japonica* roots and *M. pyrifera* at pH 7.0 or 8.0. **, ns: significant at *p* < 0.01, or not significant, respectively. The data presented in the graphs are from triplicate experiments (mean ± SD).

For the degradation of *L. japonica* root powder, the optimum E/S ratio, hydrolysis time and hydrolysis temperature were determined to be 1600 U/g, 3 h and 45 °C, respectively (Figure 4A–C). AlyP18 exhibited comparable hydrolysis efficiency on *L. japonica* root powder at pH 7.0 and pH 8.0. However, a slightly higher efficiency was observed at pH 8.0, indicating that pH 8.0 is the optimal hydrolysis pH of *L. japonica* root powder (Figure 4D). In the case of *M. pyrifera* powder degradation, the optimum E/S ratio, hydrolysis time and hydrolysis temperature were 3200 U/g, 5 h and 40 °C, respectively (Figure 4A–C). Notably, while pH 7.0 or pH 8.0 exhibited minimal effects on the degradation of *L. japonica* roots, it had a significant effect on the degradation of *M. pyrifera* powder (Figure 4D). Adjusting pH from 7.0 to 8.0 resulted in a 10.05% increase in hydrolysis efficiency of *M. pyrifera* powder (Figure 4D). Therefore, the optimal pH for hydrolyzing *M. pyrifera* powder was also pH 8.0. Many alginate lyases screened from brown algae exhibit an optimal pH of 8.0, such as AlyC8 from *Sargassum* and AlyC3 from *Laminaria* [28,35]. However, the optimal pH for the hydrolysis of brown algae by AlyP18 was slightly different from that for the degradation of pure sodium alginate, which may be due to the effect of various other components like ions, proteins and lipids in brown algae in addition to alginate [36]. After optimization, the maximum hydrolysis efficiency of *L. japonica* root powder and *M. pyrifera* powder reached 53% and 77%, respectively. Since the hydrolysate also contains some other substances besides AOs, the sulfamate/m-hydroxydiphenyl method was employed for the accurate determination of AO production, considering that uronic acids in brown algae are mainly present in alginate [37,38]. The AO production was determined to be ~320 mg/g in *L. japonica* roots and ~465 mg/g in *M. pyrifera*. As reported, the M/G of alginate in *Laminaria* roots is lower than that in *M. pyrifera* [39], indicating that the alginate in *M. pyrifera* contains more M monomers. Although AlyP18 was a bifunctional enzyme, its activity towards PM was greater (Figure 2B), which may lead to a more thorough degradation of alginate in *M. pyrifera*, resulting in higher AO production in *M. pyrifera* compared to *L. japonica roots*. The AO production corresponded to the alginate content (~40%) in brown algae [7], which indicated that AlyP18 efficiently degraded the alginate in both *L. japonica* roots and *M. pyrifera* into AOs, suggesting its effectiveness in decomposing diverse brown algae.

### 2.5. Analysis of the Degradation Products Generated by AlyP18 from L. japonica Roots and M. pyrifera

**Figure 5:**
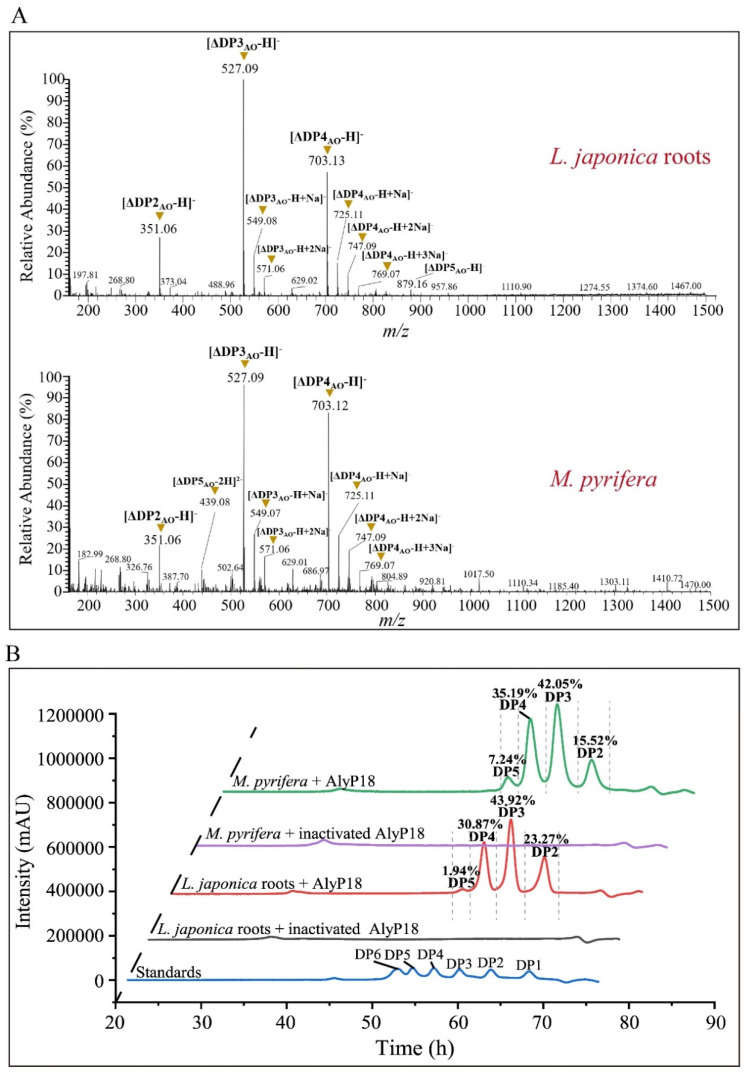
The degradation products of AlyP18 from *L. japonica* roots and *M. pyrifera*. (**A**) LTQ−Orbitrap−MS spectrum of the degradation products. AO, alginate oligosaccharide. (**B**) HPLC analysis of the degradation products. The standards used were mannuronate oligomers ranging from DP1 to DP6. DP, degree of polymerization. The figures are representatives of three experimental repeats.

The hydrolysate composition of *L. japonica* roots and *M. pyrifera* by AlyP18 was analyzed by LTQ-Orbitrap-MS and high-performance liquid chromatography (HPLC). LTQ-Orbitrap-MS results indicated that the main peaks in the hydrolysates of *L. japonica* roots and *M. pyrifera* corresponded to unsaturated alginate disaccharides, trisaccharides, tetrasaccharides and pentasaccharides (Figure 5A). Among these, alginate trisaccharides were the most abundant in both hydrolysates, accounting for 44% and 42% of the total AO production from *L. japonica* roots and *M. pyrifera*, respectively. Alginate tetrasaccharides accounted for 31% of the AOs produced from *L. japonica* roots and 35% from *M. pyrifera*, making them the second most abundant. Alginate disaccharides accounted for 23% of *L. japonica* roots and 16% of *M. pyrifera*, ranking third in abundance. Alginate pentasaccharides were the least abundant (Figure 5B). AlyP18 hydrolyzed two different brown algae substrates and produced similar AO products, implying the potential of AlyP18 in the preparation of AOs with DP2−DP5 from different brown algae. Previous studies have demonstrated that AOs with different DPs exhibit distinct bioactivities [15,40,41]. AOs with DP2–DP5 have been extensively investigated and found to induce nitric oxide production in RAW264.7 cells, enhance the drought stress resistance of tomato seedlings and improve the non-specific immune response of sea cucumber coelomocytes [42,43,44]. Therefore, AOs produced by AlyP18 may have considerable potential in the pharmaceutical, agriculture and aquaculture fields.

To date, only two studies have reported a direct AO preparation from brown algae using alginate lyases. One was to utilize PL7 alginate lyase AlgL7 to produce AOs from *L. japonica* and the other was to use PL7 VfAly7 to prepare AOs from *U. pinnatifida* [18,19]. AlgL7 produced AOs with DP2 and DP3 from *L. japonica* after 60 h degradation [18], which were different from those produced by AlyP18, indicating that the two AO products may have different biological activities. VfAly7 produced AOs with DP2-DP5 from *U. pinnatifida* after 12 h-degradation with an E/S ratio of 300 U/g (enzyme activity measured by the DNS method), and the AO production was 209 mg/g *U. pinnatifida* [19]. In comparison, AlyP18 required only a 3–5 h degradation of the algae. The E/S ratios used for the degradation of *L. japonica* roots and *M. pyrifera* by AlyP18 were 24 U/g and 48 U/g, respectively, when the enzyme activity was also determined by the DNS method. Moreover, the AO production reached ~320 mg/g in *L. japonica* roots and ~465 mg/g in *M. pyrifera*. Thus, compared with VfAly7, the use of AlyP18 results in the production of a greater quantity of AOs with a smaller amount of enzyme and shorter hydrolysis time, leading to significant cost savings and enhanced economic benefits. In addition, the limitations of the AO preparation are reflected not only in the available alginate lyase but also in the brown algae feedstock. No alginate lyases have been reported to produce AOs from *L. japonica* roots and *M. pyrifera*. The utilization of the underutilized *L. japonica* roots in the food industry and abundant *M. pyrifera* for AO preparation represents a high-value utilization of marine algae resources, which is of great significance.

## 3. Conclusions

In this study, an efficient PL18 alginate lyase AlyP18 was obtained by analyzing the secretome of the algae-decomposing strain *Pseudoalteromonas agarivorans* A3. The recombinant full-length AlyP18, consisting of two domains (ND and CD), was a mesothermal, salt-tolerant, endolytic and bifunctional alginate lyase, displaying both high enzymatic activity and substrate affinity. To assess the feasibility of utilizing AlyP18 to directly degrade brown algae for AO preparation, underutilized *L. japonica* roots in the food industry and abundantly available *M. pyrifera* from the ocean were chosen as the raw materials. Through optimization of the hydrolysis parameters of AlyP18 on *L. japonica* roots and *M. pyrifera*, the hydrolysis efficiency reached 53% and 77%, respectively. The AO production from *L. japonica* roots and *M. pyrifera* was ~32% and 46.5%. The generated AOs comprised dimers to pentamers, with trimers and tetramers being predominant. These results suggest that the alginate lyase AlyP18 is a potent tool in preparing AOs by directly decomposing brown algae, and the AOs produced by AlyP18 may have promising applications in pharmaceutical, agriculture and aquaculture fields. Additionally, the observed differences in the degradation efficiency of AlyP18 on the two different algae species may suggest the need for a detailed evaluation of the substrate specificity of alginate lyases, particularly regarding the composition of alginate monomers in the algae, before industrial application. This will help in making a more targeted selection of the enzyme.

## 4. Materials and Methods

### 4.1. Materials and Strains

The sun-dried *L. japonica* roots were purchased from the local seafood market, washed, dried, milled into powder and stored at room temperature after being sieved through an 80-mesh sieve. *M. pyrifera* powder was kindly presented by Qingdao Zhongda Agritech Co., Ltd. (Qingdao, China). Sodium alginate and glucose were purchased from Sinopharm Co., Ltd. (Shanghai, China). PM, PG and mannuronate oligomers were purchased from Zzstandard (Shanghai, China). PMG was prepared according to the method described previously [45]. D-glucuronic acid was purchased from Sigma (Saint Louis, MO, USA). *P. agarivorans* A3, previously isolated from a *L. japonica* sample, was preserved in our lab.

### 4.2. Secretome Analysis

Strain A3 was cultured following the optimal conditions for alginate lyase production reported by Sun et al. [20]. The culture was centrifuged at 12,000 × *g* for 30 min, and the obtained supernatant was then processed according to the method described previously [46]. Briefly, the proteins in the supernatant were initially precipitated by acetone solution and then denatured, reduced, alkylated and digested with trypsin. After digestion, the resultant peptides were desalted and analyzed by LC-MS/MS. The results were imported into Proteome Discoverer software 2.3 (Thermo Scientific, Waltham, MA, USA) with the Sequest HT search engine against the genome-annotated protein database of strain A3 for data processing. The secretome data were submitted to the ProteomeXchange Consortium through the iProX partner repository [47] with the dataset identifier PXD037880.

### 4.3. Bioinformatics Analysis

The putative alginate lyases of strain A3 were predicted using the dbCAN meta server (https://bcb.unl.edu/dbCAN2/blast.php, accessed on 25 September 2024) based on the genome of *P. agarivorans* A3 (GenBank: JAPCKL000000000.1) [48]. Signal peptide prediction was performed using the SignalP 6.0 Server (https://services.healthtech.dtu.dk/services/SignalP-6.0/, accessed on 25 September 2024) [49]. The conserved domains were analyzed in the NCBI’s Conserved Domain Database [50]. The 3D structure prediction of AlyP18 was executed by using Alphafold2 [51].

### 4.4. Gene Cloning, Protein Expression and Purification

The *alyP18* gene (GenBank: WP_004587971.1), excluding the predicted signal peptide, was amplified from the genomic DNA of strain A3 and inserted into the vector pET-22b between the *Nde*I and *Xho*I sites. The constructed plasmid was then transformed into *E. coli* BL21 (DE3) cells, followed by induction for protein expression with 0.3 mM isopropyl β-D-thiogalactopyranoside (IPTG) at 18 °C. The recombinant AlyP18 was purified by Ni^2+^-nitrilotriacetic acid resin (Qiagen, Dusseldorf, Germany) and then desalted on a desalination column (Cytiva, Marlborough, MA, USA).

### 4.5. Alginate Lyase Assay

The alginate lyase activity of AlyP18 was determined by the ultraviolet absorption method [28] unless otherwise stated. Briefly, the reaction was conducted in a 200 μL mixture containing 7.5 nM AlyP18, 2 mg/mL substrate and 0.5 M NaCl in 50 mM Tris-HCl (pH 7.0) at 50 °C for 15 min. The increase in absorbance at 235 nm of the mixture was monitored using a Jasco V-550 spectrophotometer (Jasco, Tokyo, Japan). One unit (U) of enzyme activity was defined as the amount of enzyme required to cause an increase of 0.1 at 235 nm per min.

The alginate lyase activity of AlyP18 was also measured by the DNS method, as described previously by Sun et al. [20]. The reaction system containing 2 mg/mL sodium alginate, 37.5 nM AlyP18, 0.5 M NaCl and 50 mM Tris-HCl (pH 7.0) was incubated at 50 °C for 15 min. One unit (U) of enzyme activity was defined as the amount of enzyme required to release 1 µmol reducing sugars (glucose equivalent) per min.

### 4.6. Biochemical Characterization of AlyP18

The substrate specificity of AlyP18 was assessed using sodium alginate, PM, PG and PMG as substrates. The optimum reaction conditions of AlyP18 were analyzed with sodium alginate. AlyP18’s optimum temperature for activity was investigated within a temperature range of 10 to 70 °C in 50 mM Tris-HCl (pH 7.0) containing 0.5 M NaCl. The optimum pH for AlyP18 activity was measured at 50 °C in the Britton–Robinson buffer spanning pH 5.0 to 10.0, which was prepared following the method described by Wang et al. [52]. The effect of NaCl concentrations on AlyP18 activity was examined over a range of 0 to 3.0 M. The kinetic parameters of AlyP18 were determined by measuring enzyme activity towards sodium alginate at various concentrations (0.1–3 mg/mL) under the optimum reaction conditions and analyzed by the Michaelis–Menten equation using the Origin 8.5 software [53].

The degradation pattern of AlyP18 was investigated towards sodium alginate. The degradation reaction, with concentrations of 2.5 μM AlyP18 and 2 mg/mL substrate, was conducted under optimum reaction conditions for 2 min, 5 min, 15 min, 1 h or 12 h. The reactions were terminated by boiling for 10 min, and the degradation products were then analyzed using HPLC on a Superdex Peptide 10/300 GL column (GE Healthcare). NH_4_HCO_3_ at a concentration of 0.2 M was taken as the running buffer with a flow rate of 0.3 mL/min. Elution was monitored at 210 nm by a UV detector. Online monitoring and data analysis were conducted using LabSolutions software, version 5.92 [54]. The final degradation products of AlyP18 toward PM, PG, PMG and sodium alginate were also analyzed by HPLC using the method described above. The reaction mixture containing 2.5 μM AlyP18 and 2 mg/mL substrate was carried out under the optimum reaction conditions for 12 h.

### 4.7. Optimization of the Hydrolysis Parameters of AlyP18 on L. japonica Roots and M. pyrifera

To ascertain the optimal E/S ratio, 20 mg/mL *L. japonica* root powder or *M. pyrifera* powder was reacted with AlyP18 at 50 °C and pH 7.0 for 12 h with various E/S ratios (0, 3, 10, 20, 40, 100, 400, 800, 1600, 2400, 3200 or 4000 U/g for *L. japonica* roots and 0, 20, 40, 100, 400, 800, 1600, 2400, 3200 or 4000 U/g for *M. pyrifera*). The optimum hydrolysis time was determined by hydrolyzing *L. japonica* root powder or *M. pyrifera* powder with AlyP18 at 50 °C and pH 7.0 for various times (0, 0.083, 0.167, 0.5, 1, 2, 3, 4, 5 or 6 h) with an E/S ratio of 1600 U/g for *L. japonica* roots and 3200 U/g for *M. pyrifera*. The optimal hydrolysis temperature was determined by hydrolyzing *L. japonica* root powder or *M. pyrifera* powder with AlyP18 at pH 7.0 with an E/S ratio of 1600 U/g for 3 h or 3200 U/g for 5 h, respectively, at different temperatures (10, 20, 30, 40, 45, 50, 60 or 70 °C). The hydrolysis experiments on *L. japonica* root powder and *M. pyrifera* powder at pH 7.0 or 8.0 were conducted under the optimal E/S ratio, hydrolysis time and hydrolysis temperature. The reaction mixture was centrifuged at 12,000 × *g* for 15 min after hydrolysis. After centrifugation, the supernatant was collected as the hydrolysate, and the precipitated *L. japonica* root powder or *M. pyrifera* powder was freeze dried and weighed. The hydrolysis efficiency was calculated by the following equation:

Hydrolysis efficiency (%) = (W_a_ − W_b_)/W_a_ × 100 (1)

where W_a_ and W_b_ are the weight of the samples before and after being hydrolyzed.

### 4.8. Degradation Product Analysis of L. japonica Roots and M. pyrifera

The hydrolysate was filtered through a 3 kDa ultrafiltration tube to remove soluble polysaccharides with high molecular mass. After ultrafiltration, the uronic acid content in the hydrolysate was analyzed by the sulfamate/m-hydroxydiphenyl assay with D-glucuronic acid as the standard [37]. The degradation products of *L. japonica* roots or *M. pyrifera* were identified by high-resolution LTQ-Orbitrap-MS (Thermo, Waltham, MA, USA) from 160 to 1500 *m*/*z* in the negative-ion mode. AO composition in the *L. japonica* roots or *M. pyrifera* hydrolysate produced by AlyP18 was analyzed by HPLC [54]. The proportion of each AO was calculated according to the percentage of the AO peak area in the chromatogram.

## Figures and Tables

**Table 1:** Secretome analysis of the extracellular alginate lyases of strain A3 cultivated under the optimum conditions for alginate lyase production.

Name	Family	Locus Tag	Signal Peptide	PSMs	Abundance ^a^
AlyP18	PL18	OIZ54_07305	Yes	155	95.09%
AlyP6	PL6	OIZ54_09025	Yes	5	3.07%
AlyP7	PL7	OIZ54_12210	Yes	3	1.84%

^a^ Abundance was calculated according to the proportion of the PSMs of an alginate lyase in the sum of PSMs of all alginate lyases in the secretome.

## Data Availability

The data in this study are contained within the article; further inquiries can be directed to the corresponding author.
